# Importancia de la tomografía computarizada de haz cónico en el reconocimiento de la trayectoria y sus variantes anatómicas del canal mandibular. Una revisión de la literatura

**DOI:** 10.21142/2523-2754-0901-2021-046

**Published:** 2021-03-11

**Authors:** Heddiksson Mario Cajahuanca Igreda, Jhoana Mercedes Llaguno Rubio, Paola Elena Medina Ocampo

**Affiliations:** 1 División de Ortodoncia, Universidad FAIPE. Cuiabá, Brasil. heddik.29@gmail.com División de Ortodoncia Universidad FAIPE Cuiabá Brasil heddik.29@gmail.com; 2 División de Radiología Bucal y Maxilofacial, Universidad Científica del Sur. Lima, Perú. académico@ilaeperu.com Universidad Científica del Sur División de Radiología Bucal y Maxilofacial Universidad Científica del Sur Lima Peru académico@ilaeperu.com; 3 Facultad de Odontología, Universidad Mayor de San Andrés. La Paz, Bolivia. paomedinaocampo@gmail.com Universidad Mayor de San Andrés Facultad de Odontología Universidad Mayor de San Andrés La Paz Bolivia paomedinaocampo@gmail.com

**Keywords:** canal mandibular, canal mandibular bífido, canal mandibular trífido, tomografía computarizada de haz cónico, mandibular canal, bifid mandibular canal, trifid mandibular canal, cone beam computed tomography

## Abstract

El objetivo de este estudio fue realizar una revisión actualizada de la literatura sobre la importancia del uso de la tomografía computarizada de haz cónico (TCHC) en el reconocimiento de la trayectoria y las variantes del canal mandibular (VCM), ya que esta permite obtener imágenes de alta calidad, con una exactitud de 94%, aproximadamente, mientras que la radiografía intraoral periapical (RIP) tiene un 53% y la radiografía extraoral panorámica (REP) presenta un 17% de exactitud. Las incidencias de las variantes del canal mandibular en estudios realizados utilizando TCHC fueron entre un 1,3% y un 69%. Estas pueden diferir entre los pacientes de diferentes orígenes étnicos y, a su vez, dentro de la misma población étnica; además, hay grandes diferencias en los tipos y configuraciones de las VCM dentro de cada grupo étnico. Estudios realizados han demostrado histológicamente el contenido de las VCM; la presencia de haces de nervios y arterias de diferentes calibres sugieren también que los pacientes presentan síntomas clínicos solamente si el paquete neurovascular alcanza cierto tamaño y número de fascículos. En este estudio se describieron las diferentes clasificaciones realizadas y actualizadas con TCHC.

## Abstract (en)

The objective of this study was to provide an updated review of the literature on the importance of the use of cone beam computed tomography (CBCT) in the recognition of the trajectory and variants of the mandibular canal (MCV).CBCT allows obtaining high quality images and visualization with an accuracy of approximately 94%, compared to 53% with periapical intraoral radiography (RIP) and 17% with panoramic extraoral radiography (REP), making CBCT an important diagnostic tool.The incidences of MCV in CBCT studies were between 1.3% and 69%, with differences between patients of different ethnic origins and within the same ethnic population, and in the types and configurations of MCV within each ethnic group. The studies available in the literature provide a histological description of the content of MCV. The presence of nerve and artery bundles of different calibers suggests that patients present clinical symptoms only if the neurovascular bundle reaches a certain size and number of fascicles. This review provides a description of the different classifications available and updated with CBCT.

## INTRODUCCIÓN

El examen imagenológico es un pilar fundamental para un correcto diagnóstico y la planificación de un tratamiento. En la amplia gama de exámenes radiológicos, la TCHC permite obtener imágenes en los tres planos del espacio: coronal, sagital y transverso o axial, lo que facilita una evaluación tridimensional de los elementos anatómicos del macizo facial y las variantes anatómicas que pudieran presentarse ^1^^-^^3^.

Una de las estructuras a tener en cuenta en la mandíbula antes de realizar tratamientos en odontología es el canal mandibular (CM), debido a las variaciones estructurales y de trayectoria que puede presentar. Este comienza desde la cara medial de la rama mandibular, a partir del agujero mandibular, y se extiende inferior y anteriormente describiendo una curva anterior y superior. Puede estar bien delimitado por paredes o describir un trayecto a través de las trabéculas del tejido óseo esponjoso; asimismo, se divide anteriormente en un conducto mentoniano y otro incisivo. El nervio mentoniano emerge por el agujero del mismo nombre y el nervio incisivo continúa su trayecto anteriormente, a través de las celdas de tejido óseo esponjoso ^4^.

Las variaciones del canal mandibular se pueden explicar durante el desarrollo embrionario, en la séptima semana, aproximadamente. Durante este periodo, el nervio alveolar inferior se presenta como tres ramos separados, los cuales inervarán a tres grupos de dientes mandibulares. El primero inerva los incisivos temporales; el segundo, los molares temporales, y el tercero, los molares permanentes. Durante el crecimiento prenatal, estos grupos de nervios se fusionan para formar el nervio alveolar inferior único; su incompleta fusión daría lugar a la formación de canales mandibulares accesorios ^1^^,^^5^^,^^6^. 

Radiográficamente, el CM puede describirse como una cinta o banda radiolúcida, entre dos líneas radiopacas paralelas, finas, conocidas como vías de tren. También lo describen como una sombra lineal oscura con delgados bordes radiopacos, superior e inferior, proyectados por lámina del hueso que limita al CM ^(6, 7)^. Las radiografías extraorales panorámicas (REP) son imágenes bidimen-sionales que no permiten la evaluación en sentido bucal-lingual ni sagital, lo que es importante durante la valoración prequirúrgica. Asimismo, algunas otras desventajas de esta técnica por imagen son la posición del paciente, la distorsión por aumento y la superposición de las estructuras anatómicas, lo que nos brinda información limitada o incluso engañosa ^8^^-^^15^. Por otro lado, la TCHC proporciona imágenes tridimensionales claras y de alta precisión que permiten visualizar la anatomía del CM y sus variaciones ^15^^-^^23^.

Diferentes estudios han comparado las REP y la TCHC en la evaluación y la incidencia de las VCM, y han demostrado los beneficios de la TCHC en el hallazgo de variantes ^8^^,^^24^^-^^26^. Sin embargo, es importante mencionar que existen también estudios que indican que la diferencia entre ambas técnicas imagenológicas en la evaluación e incidencia de las VCM no es significativa ^27^^,^^29^.

Existen diferentes clasificaciones de las VCM descritas en la literatura, todas ellas obtenidas de estudios realizados mediante REP. La primera clasificación realizada en TCHC fue descrita en el 2009 y consideró 4 tipos: canal retromolar, canal dental, canal con o sin confluencia anterior, y canal bucal-lingual ^1^.

El reconocer la localización, la configuración y las variaciones anatómicas del CM es importante para realizar procedimientos odontológicos a nivel de la mandíbula, como la colocación de implantes oseointegrados, extracciones de terceros molares, endodoncias, osteotomías sagitales de la rama y otras cirugías bucales, además de la realización de técnicas anestésicas ^(4, 28-31)^. Todo procedimiento quirúrgico realizado en el sector posteroinferior de la mandíbula requiere particular conocimiento de la posición y trayectoria del CM, pues lo contrario puede afectar el éxito de los diferentes procedimientos quirúrgicos, complicar y alargar tratamientos, lo que genera repercusiones de tipo ético y médico-legal ^2^.

El propósito de esta revisión de la literatura es describir la importancia del uso de la tomografía computarizada de haz cónico en la identificación, la configuración anatómica, la trayectoria normal y las variantes anató-micas del canal mandibular, para la correcta planificación y la realización de procedimientos odontológicos en el maxilar inferior.

## MATERIALES Y MÉTODOS

La revisión de la literatura se realizó mediante artículos con referencias al tema, los cuales fueron obtenidos de las bases de datos de Medline vía PubMed, Scopus (hasta el 31 de julio del 2020) y de las revistas científicas sobre salud más importantes del área en la actualidad, como Oral Surgery Oral Medicine Oral Pathology and Oral Radiology, Oral Radilogy, Dentomaxillofacial Radiology y Journal of Oral Maxillofacial Radiology.

### Importancia de la tomografía computarizada de haz cónico en el reconocimiento de la trayectoria del canal mandibular

En 1927 se describió en un estudio que, en el 60% de los casos, el CM es un túnel bien corticalizado y, en el 40%, este carece de cortical y es solo un pasaje en el trabeculado óseo ^32^. En 1971 se describieron tres tipos de posición en referencia al canal mandibular: tipo I, canal mandibular muy cerca de las raíces dentarias; tipo II, canal mandibular situado inferior a las molares; y tipo III, canal mandibular más posterior e inferior que los anteriores tipos ^31^^,^^32^.

Con referencia a las piezas dentarias, el canal mandibular se localiza más próximo a la tabla lingual y media a nivel de las raíces de los segundos y terceros molares, subyacente al ápice del primer molar, y se vestibulariza a nivel del segundo premolar. Al observarlo por secciones, puede presentar una forma circular, ovalada o piriforme. Su calibre es mayor de posterior a anterior durante su trayectoria ^2^.

La frecuencia de las VCM era del 1% a través de las REP en diversos estudios realizados ^33^^,^^34^. Esto puede deberse a la calidad de las imágenes, que era muy inferior a las panorámicas de la actualidad, los métodos de análisis, la experiencia del odontólogo y los diferentes orígenes étnicos evaluados. En otros estudios, la incidencia de canales mandibulares bífidos evaluados con REP fue de un 6,4% a un 7,4% ^29^^,^^33^. 

La TCHC, introducida en 1999, brindó a los radiólogos maxilofaciales un equipo diseñado específicamente para el área del macizo facial, con grandes ventajas en comparación con la tomografía computarizada y otros exámenes radiográficos, las cuales mencionan imágenes tridimensionales claras y de alta precisión, muestra de artefactos mínimos, costo reducido, fácil manejo y niveles de dosis de radiación menores que los protocolos estándar de tomografías computarizadas. Las limitaciones de la TCHC que refieren son la falta de datos sobre los tejidos blandos y el volumen de la imagen limitado. 

Al evaluar la trayectoria intraósea del canal mandibular en sentido vestibulolingual e inferosuperior con respecto al maxilar inferior, pueden presentarse numerosas variaciones según el sexo, la edad, el origen étnico y la reabsorción ósea, por lo que se recomienda realizar un estudio imagenológico que permita una fácil visualización del canal mandibular, como la TCHC que, mediante su *software*, permite valorar de manera más precisa la posición anatómica, resaltar la ubicación y mejorar el contraste, el brillo y la escala de grises para una mejor visualización del canal mandibular ^1^^,^^6^^,^^33^^,^^35^.

En un estudio realizado en el 2010 ^2^ se utilizó la TCHC en una población de Colombia para determinar la ubicación y la trayectoria del CM. Destacó su ayuda diagnóstica altamente sensible y, entre los resultados obtenidos con este examen, se describió también que la distancia del CM a la tabla lingual es menor que al borde basal y a la tabla vestibular; asimismo, que el agujero o foramen mentoniano presenta su emergencia en un 70% apical al segundo premolar o mesial a este. La correcta ubicación del CM resulta de gran utilidad para la planificación de cualquier cirugía en la región mandibular, pues disminuye el riesgo de un trauma al nervio alveolar inferior.

Otro estudio determinó la fiabilidad de la TCHC para localizar el canal mandibular, medir su diámetro y la relación con la tabla ósea vestibular en cadáveres frescos, en comparación con medidas reales, al realizar un procedimiento quirúrgico de lateralización del nervio alveolar inferior. Los resultados mostraron una discrepancia entre las medidas obtenidas con la TCHC, que fueron mayores que las medidas reales (1,15 mm con relación al canal y la tabla ósea vestibular, y 0,30 mm con relación al grosor del canal mandibular. Es clara la importancia de conocer esta discrepancia debido a los diferentes procedimientos quirúrgicos que se pueden realizar en esa zona y están cercanos al nervio alveolar inferior ^42^.

### Importancia de la tomografía computarizada de haz cónico en el reconocimiento de las variaciones anatómicas

Las VCM se conocen con el nombre de accesorios, bífidos o trífidos. El canal mandibular bífido se presenta como una hendidura en dos partes o ramas, en la que se observa una división, cada una con su propio canal, cada uno de los cuales puede contener un haz neurovascular en diferentes formas. Esta variación puede presentarse tanto unilateral como bilateralmente ^36^^,^^39^. En estudios realizados utilizando la TCHC, su incidencia fue entre un 1,3% y un 69% ^6^^,^^30^.

En un estudio se examinaron mandíbulas de humanos disecados y se mencionan tres tipos de disposición del nervio alveolar inferior: el tipo I, en el que el nervio dentario inferior es único y de gran estructura, acostado en un canal óseo; el tipo II, en el cual el nervio dentario inferior se encuentra reducido sustancialmente hacia abajo en la mandíbula; y el tipo III, en el que el canal se separa posteriormente en dos ramas grandes que, en conjunto, se pueden considerarse equivalente a una rama alveolar ^31^.

Otro estudio describió tres patrones principales de duplicación. El tipo I presenta canales duplicados originarios de un solo agujero mandibular, por lo general del mismo tamaño, y es el más común. Puede presentarse de dos maneras: el tipo IA, donde el canal inferior es a veces más pequeño, y el tipo IB, donde el canal superior es el más pequeño. El tipo II presenta un canal superior corto que se extiende al segundo, al tercero o a la zona del tercer molar. El tipo III presenta dos canales de igual tamaño derivados de forámenes separados que se unen en la zona molar y es el menos común. El tipo IV es una variación de dos canales, en el cual los canales suplementarios se deben a la zona retromolar y se unen a los canales principales en las áreas retromolares.

También se clasificó según su localización anatómica y configuración, y se describieron cuatro tipos. El tipo I representa canales bífidos bilaterales o unilaterales que se extienden a la zona del tercer molar o al área inmediatamente circundante; el tipo II presenta canales bífidos bilaterales o unilaterales que reúnen dentro de la rama de la mandíbula; el tipo III es una combinación de los tipos 1 y 2; y el tipo IV son dos canales, cada uno de los cuales se origina a partir de un foramen mandibular y se unen para formar una canal más grande. Además, indicaron que los canales normales o los canales bífidos pueden tener canales adicionales más pequeños ^1^^,^^6^^,^^33^^,^^35^.

En el 2009 se realizó la primera clasiﬁcación del canal mandibular bífido (CMB) utilizando TCHC y se describieron cuatro tipos: el tipo 1 o canal retromolar, que presenta una bifurcación en la región de la rama mandibular, no alcanza piezas dentales y realiza una curva para llegar a la región retromolar; el tipo 2 o canal dental, bifurcada y que alcanza el ápice de la raíz del segundo o tercer molar; el tipo 3, con y sin conﬂuencia anterior, el canal mandibular presenta una bifurcación que continúa su recorrido hacia el sector anterior, luego puede volverse unirse o no con el canal mandibular principal; y el tipo 4 o canal bucal-lingual, que presenta una bifurcación hacia bucal o lingual con respecto al canal principal ^1^^,^^6^.

En el 2014 se añade un tipo o categoría llamando canal superior, que presentaba una bifurcación y recorría hacia arriba, y que no cumplía con los criterios de clasificación del 2009 ^26^. En el 2018 se sumó otra categoría, el canal de rama, a la clasificación de CMB, mediante el uso de TCHC ^31^.

El primer caso de canal mandibular trífido (CMT) se informó en 2005, siendo la menos estudiada hasta el momento. En el 2010, se informó de 5 prominentes canales mandibulares accesorios bilaterales. Un año después se reportó un caso raro de un conducto accesorio en un paciente con canal mandibular trífido, que incidió después de la inserción del implante, lo que le provocó malestar y dolor. El CMT ha sido reportado como varias combinaciones de bifurcaciones del canal mandibular, como dos canales anteriores, dos canales retromolares o canales dentales. En el 2014 se propuso una clasificación de TCM en 5 subtipos; el primero son dos canales accesorios del tipo retromolar; el segundo, dos canales accesorios uno retromolar y uno dental; el tercero, dos canales accesorios del tipo dental; el cuarto, dos canales accesorios, uno dental y uno hacia delante; el quinto, dos canales retromolares accesorios con dos agujeros inferiores. En los años 2017 y 2018 se utilizaron descripciones narrativas de la clasificación de CMB mediante TCHC del 2009 para describir los canal mandibulares trífidos observados ^31^.

En el 2011 ^31^, informaron de la separación intencional de los paquetes neurovasculares accesorios en 4 casos de cirugía periapical de los premolares inferiores; uno de los casos sufrió parestesia posoperatoria. Al realizar el examen histopatológico, resultó contener entre uno y cuatro fascículos nerviosos; el caso de parestesia tenía cuatro fascículos, lo cual sugería que los síntomas clínicos se presentan solamente si el paquete neurovascular alcanza un cierto tamaño y número de fascículos. Otro estudio, también realizado en el 2011 ^36^, informó la presencia de un canal mandibular bífido en una cirugía de tercer molar, lo enviaron a examen histopatológico y se confirmó ser tejido nervioso. 

**Figura 1:**
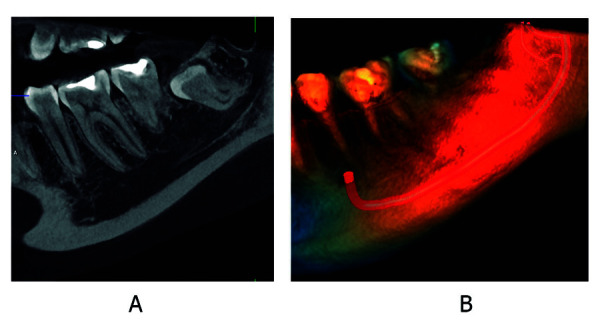
Canal mandibular trífido, dos canales retromolares. A. Corte sagital de la región de pieza dentaria 3.8 del lado izquierdo mediante TCHC. B. Renderizado de TCHC.

Un estudio de canales mandibulares bífidos bilaterales en un cadáver japonés, mediante imágenes de radiografía panorámica, tomografía computarizada helicoidal, tomografía computarizada de haz cónico, comparando con cortes anatómicos de la mandíbula y estudio histológicos de su contenido, señaló que la TCHC es valiosa para la evaluación de los canales mandibulares bífidos, la distribución del canal era más clara y fueron consistentes con las secciones anatómicas. La radiografía panorámica halló la incidencia de canal mandibular bífido en un solo lado. El estudio histológico mostró que los canales contenían varios haces de nervios y arterias, y que los más grandes eran de un tamaño similar, con lo que determinó la importancia de identificar las variaciones del canal mandibular, pues tiene implicaciones clínicas en procedimientos quirúrgicos en la zona mandibular, como implantes dentales, extracción de terceros molares impactados y osteotomía sagital de rama. Las complicaciones que se podrían presentar serian neuroma traumático, parestesia posoperatoria, sangrado durante la cirugía y lesión quirúrgica iatrogénica, lo que alarga tratamientos y tiene repercusiones de tipo ético y médico-legal ^37^ (Figuras 1 y 2).

**Figura 2:**
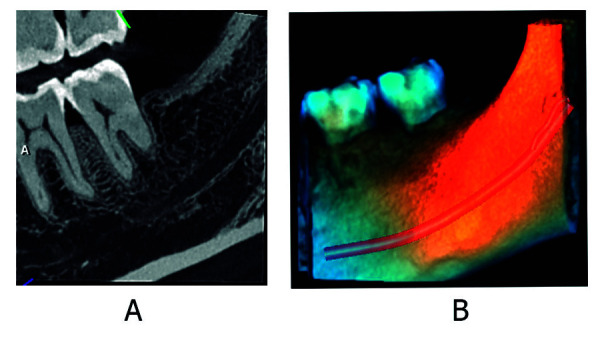
Canal mandibular bífido, tipo con confluencia anterior. A. Corte sagital de la región de pieza dentaria 3.8 ausente del lado izquierdo mediante TCHC. B. Renderizado de TCHC.

### Importancia de la tomografía computarizada de haz cónico en el reconocimiento de las variantes anatómicas y de trayectoria del canal mandibular frente a las imágenes bidimensionales

La TCHC es la ayuda diagnóstica más adecuada para la visualización del canal mandibular. La TCHC presenta un 94% de exactitud, aproximadamente, mientras que la radiografía periapical tiene un 53% y las imágenes panorámicas presentan un 17% de exactitud ^(2, 32, 42)^. Los exámenes bidimensionales, como las REP, han sido utilizados para reconocer y clasificar las VCM; sin embargo, la superposición de estructuras observadas, las diferencias de morfología mandibular, la no disposición en sentido transversal o bucolingual hacen que omita una parte importante en su orientación espacial, ya que observar estructuras tridimensionales en imágenes de dos dimensiones podría ser una fuente potencial de diagnóstico erróneo. Las radiografías convencionales como la panorámica, a diferencia de la tomografía computarizada, solo pueden sugerir el diagnóstico pero no confirmarlo ^37^.

La prevalencia de canales mandibulares bífidos con el uso de las REP ha reportado tasas de incidencia bajas (1%). Por otro lado, las falsas imágenes de canales bífidos pueden deberse a la huella del nervio milohiodeo en la superficie mandibular medial en el punto donde se divide desde el nervio alveolar inferior y se dirige al piso de la boca, así como a la inserción del músculo milohiodeo en la superficie mandibular interna. Además, puede ser difícil su identificación debido a las sombras de superposición como la faringe, las vías respiratorias, el paladar y la úvula ^6^^,^^36^.

Un estudio basado en imágenes tridimensionales sostiene que es el único medio que proporciona un diagnóstico irrefutable sobre la existencia de canales bífidos mandibulares ^37^. Sin embargo, otro estudio comparó las VCM mediante REP y TCHC, y sus resultados de incidencias de las VCM fueron del 7,4% y el 9,8% respectivamente, una diferencia no significativa entre ambos métodos, lo cual indica que, aunque la tomografía computarizada de haz cónico permite una mejor visualización de la ubicación, forma y relación con las estructuras anatómicas, las REP pueden ser utilizados en el estudio de los canales bífidos mandibulares ^27^. La diferencia de incidencia entre los estudios puede deberse a la calidad de la imagen panorámica, el método de análisis de las imágenes, el evaluador, la diferencia étnica, que pueden explicar la mayor incidencia de bifurcaciones del CM ^34^.

**Figura 3:**
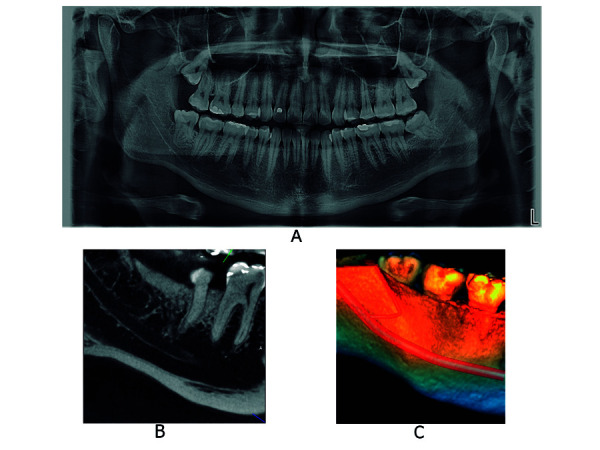
Canal mandibular bífido, tipo retromolar del lado derecho. A. Radiografía extraoral panorámica. B. Corte sagital de la región de pieza dentaria 4.8 mediante TCHC. C. Renderizado de TCHC.

**Figura 4:**
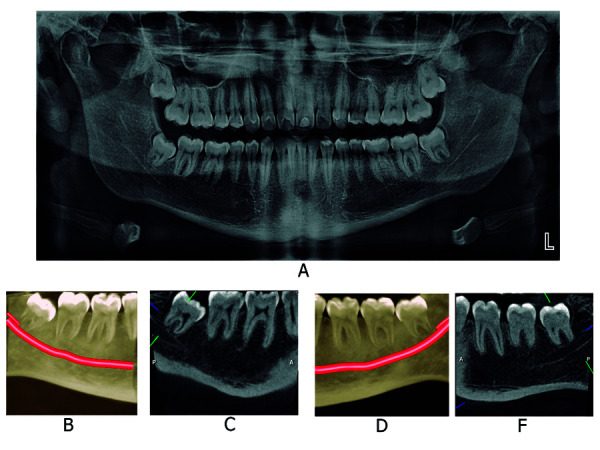
Canal mandibular bífido, tipo dental bilateral. A. Imagen extraoral panorámica. B y C. Renderizado y corte sagital de la región de pieza dentaria 4.8 del lado derecho. D y F. Renderizado y corte sagital de la región de pieza dentaria 3.8 del lado izquierdo.

Asimismo, un estudio comparó la incidencia de canales bífidos mediante el uso de tomografía computarizada, tomografía computarizada de haz cónico y radiografía extraoral panorámica. En veintiocho pacientes, la tomografía computarizada de haz cónico visualizó 19 casos y la tomografía computarizada, solo 15. Los autores señalan que ambos métodos son viables para el reconocimiento de estas variantes; no obstante, la tomografía computarizada de haz cónico proporciona mayores ventajas como tamaño de voxel menor, dosis de radiación relativamente baja, mejor calidad de imagen y equipo asequible. Además, las radiografías panorámicas dentales no identificaron tres de cada cinco canales bífidos, lo que sí se consiguió usando imágenes de tomografía computarizada multicorte ^31^ (Figuras 3, 4 y 5).

**Figura 5:**
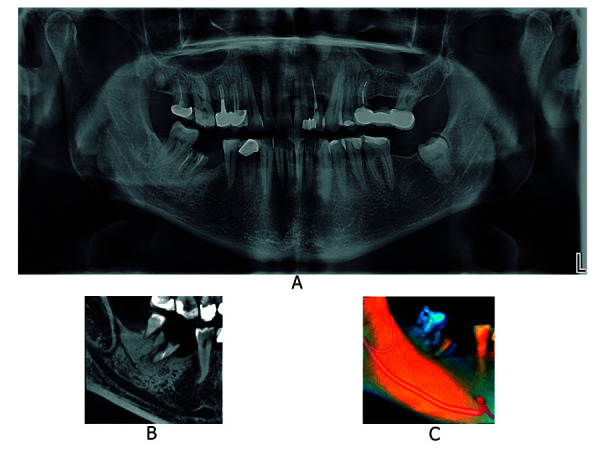
Canal mandibular con 4 canales accesorios, de tipo retromolar, tipo dental y tipo con confluencia anterior. A. Radiografía extraoral panorámica. B. Vista corte sagital de la región de pieza dentaria 4.8 mediante TCHC. C. Renderizado de TCHC.

## DISCUSIÓN

Al momento de realizar un diagnóstico y planificación de tratamiento, los odontólogos deben conocer las estructuras anatómicas normales involucradas, las variantes anatómicas y patologías que puedan presentarse en la región. Se sugiere realizar protocolos iniciales en el diagnóstico del paciente, mediante la utilización de exámenes clínicos, imagenológicos e histopatológicos. Entre la amplia gama de estudios imagenológicos, el odontólogo debe tener el conocimiento básico requerido para hacer una adecuada selección. Reconocer la localización, la configuración y las variaciones anató-micas del canal mandibular es importante para realizar procedimientos odontológicos a nivel de la mandíbula, como la colocación de implantes oseointegrados, extracciones de terceros molares, endodoncias, osteotomía sagital de la rama y otras cirugías bucales, además de técnicas anestésicas. Su desconocimiento puede afectar el éxito de los diferentes procedimientos quirúrgicos, y como consecuencia complicar y alargar tratamientos, con repercusiones de tipo ético y médico-legal.

La TCHC introducida en 1999 ayudó a los radiólogos maxilofaciales a tener un equipo diseñado específicamente para el área del macizo facial, con grandes ventajas en comparación con la tomografía computarizada y otros exámenes radiográficos. La TCHC permite visualizar una correcta ubicación, trayectoria y relación con estructuras anatómicas adyacentes al canal mandibular, por lo que resulta de gran utilidad para la planificación de cualquier cirugía en la región mandibular al disminuir el riesgo de un trauma al nervio alveolar inferior.

La primera clasificación de CMB realizada en TCHC fue descrita por Naitoh et al. ^1^^,^^6^^,^^31^ y consideró cuatro tipos: canal retromolar, canal dental, canal con o sin confluencia anterior, y canal bucal-lingual. Esta ha sido las más utilizada en la mayoría de los estudios hasta la actualidad ^1^; sin embargo, se han añadido categorías en otros estudios, como el de Muinelo Lorenzo et al. ^26^, que incluye el canal superior, y el de Moro et al. ^31^, que considera el canal de rama. 

Diversas investigaciones se han realizado mediante la utilización de TCHC y estudios histológicos, que confirmaron la presencia de haces de nervios y arterias. Von Artx et al. ^31^ sugieren la presencia de síntomas clínicos solo si el paquete neurovascular alcanza cierto tamaño y número de fascículos, lo que determina la importancia de identificar las variaciones de canal mandibular, con implicaciones clínicas, en procedimientos quirúrgicos en la zona mandibular, como implantes dentales, extracción de terceros molares impactados y osteotomía sagital de rama. Las complicaciones que se podrían presentar serían neuroma traumático, parestesia posoperatoria, sangrado durante la cirugía, lesión quirúrgica iatrogénica, que alargarían los tratamientos y generarían repercusiones de tipo ético y médico-legal.

La TCHC es la ayuda diagnóstica más adecuada para la visualización del canal mandibular, al presentar, aproximadamente, un 94% de exactitud, mientras que la radiografía periapical tiene un 53% y las imágenes panorámicas, un 17%. Entre las desventajas que presentan las REP están la superposición de estructuras observadas, las diferencias de morfología mandibular, la no disposición en sentido transversal o bucolingual, con lo que omiten una parte importante en su orientación espacial al observar estructuras tridimensionales en imágenes de dos dimensiones, lo puede ser una fuente potencial de diagnóstico erróneo. A diferencia de la TCHC, las radiografías panorámicas no permiten confirmar la presencia de variantes del canal mandibular. La mayoría de los estudios coinciden en que la TCHC es el estudio imagenológico ideal para la visualización del CM y las VCM, y solo dos estudios, el de Neves et al. ^27^ y el de Keith Sonneveld et al. ^30^, indican diferencias no significativas entre la TCHC y las REP.

La TCHC es el estudio imagenológico ideal para la visualización del CM y sus variantes anatómicas al proporcionar una mayor exactitud con respecto a otras técnicas. El odontólogo debe reconocer la localización, la configuración y las variaciones anatómicas del canal mandibular, que son importantes para realizar procedimientos odontológicos a nivel de la mandíbula. Su desconocimiento puede afectar el éxito de los diferentes procedimientos quirúrgicos, y como consecuencia complicar y alargar tratamientos, con repercusiones de tipo ético y médico-legal.

La mayoría de los métodos de formación de imágenes no pueden confirmar la presencia de estructuras intracanal, tales como nervios o vasos, a menos que se utilice la resonancia magnética (RM). En la actualidad hay muy pocas investigaciones que hayan estudiado el CM mediante RM y solo una ha estudiado las VCM con RM. Wamasing et al. ^40^, en su trabajo, utilizaron RM, TC y REP para la identificación de las VCM. Como resultado obtuvieron que la incidencia de las VCM fue del 6,4% mediante RM, la REP no pudo identificarlas y la TC apenas pudo identificar un solo caso, lo cual indica que la RM es el método ideal para obtener imágenes precisas.

Finalmente, se ha analizado que las VCM se han visualizado con una alta incidencia mediante la utilización de la TCHC y que el odontólogo tiene que considerarlas en la planificación de los tratamientos para los pacientes.

## CONCLUSIÓN

La evaluación del canal mandibular es de gran importancia antes de la realización de diferentes tratamientos odontológicos, no solo por su recorrido sino también por las variantes anatómicas presentes. Si bien las imágenes 2D son de ayuda en la identificación, presentan varias limitaciones como la bidimensionalidad y la superposición de estructuras. Por su parte, la TCHC ha demostrado ser el método ideal para estudiar esta estructura anatómica debido a sus ventajas como la evaluación en los tres planos del espacio, una alta calidad y exactitud en la imagen, lo que permite visualizar una correcta ubicación, trayectoria, variaciones del canal mandibular y relación con estructuras anatómicas adyacentes.

## Fuente de financiamiento:

Autofinanciado
